# Dual Pathways Coupled to Oxytocin Molecular Signals in Cultured Astrocytes

**DOI:** 10.3390/cells15100950

**Published:** 2026-05-21

**Authors:** Elisa Farsetti, Sarah Amato, Monica Averna, Elena Gatta, Diego Guidolin, Marco Pedrazzi, Laura Lori, Matilde Gnecco, Guido Maura, Luigi F. Agnati, Manuela Marcoli, Chiara Cervetto

**Affiliations:** 1 Department of Pharmacy, Section of Pharmacology and Toxicology, University of Genova, Viale Cembrano 4, 16148 Genova, Italy; elisa.farsetti@edu.unige.it (E.F.); amatosarah06@gmail.com (S.A.); matilde.gnecco@edu.unige.it (M.G.); 2 Department of Experimental Medicine, Section of Biochemistry, University of Genova, Viale Benedetto XV 1, 16132 Genova, Italy; monica.averna@unige.it (M.A.); marco.pedrazzi@unige.it (M.P.); laura.lori@edu.unige.it (L.L.); 3 DIFILAB, Department of Physics, University of Genova, Via Dodecaneso 33, 16146 Genova, Italy; elena.gatta@unige.it; 4 Department of Neuroscience, University of Padova, Via Gabelli 63, 35122 Padova, Italy; diego.guidolin@unipd.it; 5 Department of Earth, Environment and Life Sciences, University of Genova, Viale Benedetto XV 5, 16132 Genova, Italy; guido.maura@unige.it; 6 Department of Biomedical, Metabolic Sciences and Neuroscience, University of Modena and Reggio Emilia, 41121 Modena, Italy; luigi.agnati@gmail.com; 7 Interuniversity Center for the Promotion of the 3Rs Principles in Teaching and Research (Centro 3R), 56122 Pisa, Italy; 8 IRCCS Ospedale Policlinico San Martino, 16132 Genova, Italy

**Keywords:** primary astrocytes, glutamate release, intracellular calcium, biased agonists, oxytocin, atosiban, carbetocin

## Abstract

**Highlights:**

**What are the main findings?**

- Primary astrocytes express functional oxytocin receptors.
- Oxytocin induced dual responses in astrocytes.

**What is the implication of the main finding?**

- The neuropeptide oxytocin is a molecular signal which might play significant roles in the astrocyte functioning.

**Abstract:**

Oxytocin’s capacity to affect the glial cell functions is increasingly recognized. We previously reported that oxytocin could cause both excitation and inhibition of Ca^2+^ signals and glutamate release in the processes of adult rodent astrocytes. Our purpose here was to investigate oxytocin receptor expression and oxytocin effects in astrocytes. In primary cortical astrocytes, we assessed the presence of oxytocin receptors by confocal imaging, and the effects of oxytocin receptor activation on intracellular Ca^2+^ signals and glutamate release. We found that oxytocin receptors are expressed in both the soma and processes of astrocytes; oxytocin at nanomolar concentrations could induce dual responses in astrocytes, namely facilitation and inhibition of Ca^2+^ signals and glutamate release; the oxytocin facilitatory and inhibitory effects were duplicated by the biased agonists carbetocin and atosiban, respectively; and the facilitatory and the inhibitory effect were dependent on activation of a G_q_ and a G_i_ pathway, respectively. It is concluded that oxytocin effects in astrocytes could duplicate the effects in processes prepared from astrocytes matured in neuron-astrocyte networks, substantiating the use of astrocytes to study astrocytic oxytocin molecular signaling.

## 1. Introduction

Oxytocin (OT), a neuropeptide released from hypothalamic neurons, operates as a molecular signal involved in complex brain functions including social behaviors and emotionality, promoting social interactions and reproduction [1,2]. The centrally mediated effects depend on OT action upon oxytocin receptors (OTRs) distributed widespread in the brain. Until recently, OT was supposed to act almost exclusively upon neurons [3,4,5] and OT actions on glial cells were overlooked. Evidence is now accumulating that astrocytes may be involved in centrally mediated OT effects. The finding might not be unexpected if one considers how interactions between astrocytes and neurons are critical to complex brain functions. Indeed, in neuron-astrocyte networks, astrocytes can respond with membrane depolarization to neuron activity [6,7], express receptors for neurotransmitters (see [8,9,10,11]) and release gliotransmitters that act upon neuronal receptors [12]. Such bidirectional communication between the astrocytes and neurons is recognized as crucial to maintaining neuron functions in a healthy brain, while altered astrocyte function can play primary roles in neuron dysfunction [13,14]. Receptors for OT were found in astrocytes in the hypothalamus [15], auditory cortex [16], amygdala [17], striatum [18], or hippocampus [19] of rodents, and in the human frontal cortex [20]. Expression of OTR was also described in cultured astrocytes, in the soma and processes of cells that are positive for the glial fibrillary acidic protein (GFAP) in primary cultures of hypothalamic [21] or spinal astrocytes [22]. The relevance of astrocytic OTR signaling in behavior is emerging. Astrocytic OTRs in the amygdala mediated the anxiolytic and positive reinforcement effects of OT in rodents [17]; OT-induced cytoskeletal remodeling in hippocampal astrocytes affected cell interconnectivity and contributed to anxiolysis [23]. The response to acute stress appeared mediated by OTR-dependent structural and functional changes in amygdala astrocytes, with astrocytic process retraction and modulation of neuronal activity [24]. Also, at hypothalamic synapses OT was reported to affect the astrocyte coverage of the synapse, leading to morphological changes in astrocytes and neurons and modulation of the neuron-astrocyte network activity [25].

OT was reported to trigger Ca^2+^ signals in amygdala astrocytes in acute slices [17] as well as in cultured astrocytes [26,27]. Consistently, we reported that activation of OTR induced Ca^2+^ signals and glutamate release from the processes of striatal astrocytes [18,28,29,30]. Actually, we also observed that OT could evoke dual effects, namely facilitation and inhibition of the evoked Ca^2+^ signals and glutamate release from the processes [30]. These findings indicate the capacity of OTR to couple to both a stimulatory and an inhibitory pathway in the astrocyte processes [30].

Here, we investigate OTR expression and the OT effects and coupling to stimulatory and inhibitory pathways in primary cortical astrocytes. In preliminary experiments we set the optimal primary culture conditions by assessing the astrocyte morphological and functional features. An increase in intracellular cyclic adenosine monophosphate (cAMP) levels, e.g., through forskolin (FSK) activation of adenylyl cyclase, is reported to induce differentiation of astrocytes and has been used to study astrocyte functioning and gliotransmitter release [31,32,33,34]. In our hands, exposure to FSK in the culture medium induced astrocyte stellation and maturation. In FSK-exposed astrocytes, we then evaluated the effects of OT on intracellular Ca^2+^ signals and release of the gliotransmitter glutamate, also by investigating the molecular mechanisms of the receptor transduction pathways. The effect of atosiban and carbetocin, biased OTR ligands selectively activating the G_i_ and the G_q_ pathway, respectively [35,36], was also assessed.

## 2. Materials and Methods

### 2.1. Primary Astrocyte Cultures

The preparation of primary astrocyte cultures was from rat neocortices of 0- to 2-day-old Sprague Dawley pups. The pups were born in the animal facility of the Department of Pharmacy (DIFAR), University of Genova, Italy. The animals were housed in standard controlled conditions (temperature, 22 ± 1 °C; relative humidity, 50%; 12 h light/dark cycle, light 7 a.m.–7 p.m.) and had free access to a standard diet and water. Animal handling agreed with the European Communities Parliament and Council Directive 2010/63/EU and the Italian D.L. n° 26/2014, and the animal use was approved by the Italian Ministry of Health (protocol n° 75F11.N.P3C, August 2019 and 75F11.N.FIE, December 2023), in accordance with Decreto Ministeriale 116/1992. Every possible measure was taken to reduce suffering and minimize the number of animals used. Prior to decapitation, the pups were euthanised by cervical dislocation and the cerebral cortices were dissected. The cortices were enzymatically dissociated for 20 min (min) with papain (3 U/mL; Sigma Aldrich, Milan, Italy) in Dulbecco’s Modified Eagle Medium (DMEM; Gibco, Waltham, MA, USA) plus L-cysteine (1 mM; Sigma Aldrich) and DNAse I (Sigma Aldrich). The cell suspension was plated on flasks according to the original protocol [37] with some modifications. Cells were maintained for 7 days in vitro (DIV) in an atmosphere of 5% CO_2_/95% air in DMEM medium with 1 mM sodium pyruvate, 2 mM L-glutamine and supplemented with 10% fetal bovine serum (FBS; Euroclone, Milan, Italy), 1% penicillin and streptomycin (Euroclone). To obtain highly enriched astroglial cultures, at 7 DIV, sub-confluent cultures were shaken at 1800 rpm and subsequently re-plated in flasks. The induction of stellation [38,39,40] was achieved by adding FSK (10 µM; Tocris, Bio-Techne, Milan, Italy) to the culture medium, which was then maintained for a week (DMEM FSK + protocol). At 14 DIV, the cells were transferred on pre-coated glass coverslips, in cell flasks or multi-well plates, for functional or immunofluorescence assay. To maintain low contamination from microglia cells [40], the cell medium was changed regularly (at least every 2–3 days) and the cultures were trypsinized and the cells replated.

### 2.2. Intracellular [Ca^2+^] Assay

At 20–22 DIV, astrocytic [Ca^2+^]_i_ was determined as previously described on cells or subcellular particles [41]. Briefly, 1.5 × 10^4^ astrocytes were plated on black 96 multi-well plates and incubated with Calcium Green™-1 AM (CG; 10 μM) for 30 min at 37 °C in the serum free DMEM medium. The experiments were performed at 37 °C in HEPES medium (NaCl 128 mM, KCl 2.4 mM, MgSO_4_ 1.2 mM, KH_2_PO_4_ 1.2 mM, CaCl_2_ 1.0 mM, and HEPES 10 mM with glucose 10 mM). Astrocytes were exposed to the different drugs as indicated in the Figures. The assay was conducted in a resting state and in a depolarized condition using 4-aminopyridine (4-AP; 300 µM). 4-AP was here used as a stimulus capable of inducing a quasi-physiological depolarization. In fact, astrocytes express various K^+^-permeable channels [42] and 4-AP could block all the voltage-activated K^+^ channels in astrocytes, including the slowly inactivating channels implicated in resting K^+^ conductance [43]. Due to the direct blocking of voltage-gated K^+^ channels and membrane depolarization, 4-AP indirectly leads to Ca^2+^ influx by opening voltage-gated Ca^2+^ channels [44]. On the other hand, we cannot exclude that 4-AP could also directly facilitate Ca^2+^ entry through voltage-gated Ca^2+^ channels, apart from an effect on K^+^ currents, as reported in spinal neurons [45]. Indeed, culturing in our experimental conditions—in media supplemented with dibutyryl-cAMP, or FSK—seems essential for the appearance of Ca^2+^ currents in astrocytes [46]. The fluorescence-based Ca^2+^ signals were measured and analyzed as described in [30]. Briefly, using a LB940 Mithras Fluorescence Multi-Label Reader (Berthold Technologies, Baden Württemberg, Germany) in the top reading configuration, we measured the fluorescence intensities (excitation 485 nm, emission 535 nm) every 10 s for 5 min. The oscillatory nature of the Ca^2+^ signal, obtained by reading the samples every 10 s rather than continuously for 300 s, reflects the balance between Ca^2+^ transients and homeostatic recovery. Furthermore, these fluctuations are consistent with the signal-to-noise ratio typically observed in fluorescence-based kinetic assays. To standardize the measurements, the fluorescence intensity recorded at the beginning of the observation window was subtracted from each subsequent measurement. The [Ca^2+^]_i_ variation (called Delta Fluorescence) was calculated as the difference between the drug-treated and the vehicle-treated wells, as measured by the CG-dependent fluorescence every 10 s. Then, we drew the time courses of CG-dependent [Ca^2+^]_i_ variations for vehicle-treated and drug-treated wells and used them to determine the Area Under the Curve (AUC) for each condition.

### 2.3. Endogenous Glutamate Release

To assess the endogenous glutamate release, at 20–22 DIV, the cell medium was removed and substituted with fresh serum free DMEM medium. A total of 5 × 10^6^–10^7^ astrocytes were gently detached and transferred as monolayers in the lower chambers of a superfusion system. An aliquot was used for protein’s determination according to Bradford’s assay [47]. The release experiments were conducted as previously described [48] in HEPES medium maintained at 37 °C. The superfusion technique requires a constant flow (0.5 mL/min) to avoid the receptor biophase and indirect effect of any released molecule. To obtain a pharmacological characterization of the targets studied and of the involved intracellular transduction pathway, all the receptor ligands and modulators were added to the medium according to the experimental design. A peristaltic pump constantly removed the HEPES medium from the cell surfaces; the medium was renewed every 10 min. After a superfusion period necessary for the glutamate basal level stabilization, for each superfusion chamber, we collected two 3 min samples, and the mean value of the glutamate amounts in these samples was used as basal outflow. During superfusion, we collected two additional samples related to the application of the agonists OT and carbetocin, or of 4-AP (300 µM), or of the agonists (OT, carbetocin and atosiban) in the presence of 4-AP at the concentration shown in the Figures. Therefore, the agonists and/or 4-AP were applied for 6 min till the end of the experiments. The antagonist of OTR L 371,257 (0.1 µM) and the phospholipase C (PLC) inhibitor U73122 were added during superfusion, which occurred 8 min prior to the application of the agonist. In the experiments, we superfused one or more chambers with HEPES solution and/or with HEPES with added L 371,257 or U73122. The presence of L 371,257 or U73122 did not affect the basal outflow of glutamate. The amount of glutamate in the three-minute samples was determined using high-performance liquid chromatography (HPLC) as described previously [49], and then the glutamate content in each sample was expressed as pmol/mg protein. For each chamber, we calculated the glutamate overflow by subtracting two times the mean basal outflow of the chamber from the glutamate outflow measured in the additional samples of the same chamber. Then, for each experiment, we calculated the effects of the drugs as overflow; in fact, the amount of glutamate overflow in the chambers used as control was subtracted from the overflow calculated in drug-treated chambers.

### 2.4. cAMP Enzyme-Linked Immunosorbent Assay (ELISA)

At 20–22 DIV, to assess the intracellular [cAMP], 2.5 × 10^5^ cells/well were seeded in 6-multiwell plates for ELISA experiments. We used the cAMP ELISA kit (Cayman Chemical, Ann Arbor, MI, USA). The day before the experiment, the growth medium was replaced with DMEM without supplements. To increase cAMP levels, astrocytes were treated with FSK (5 µM) for 15 min at 37 °C in the absence or in the presence of OT or atosiban at the concentrations reported in the Figure. In each plate, at least a well was used for the Bradford’s protein determination. After 15 min, the medium was removed and astrocytes were lysated with 0.1 M HCl at room temperature (RT) for 20 min. Centrifugation of the lysates at 1000× *g* for 10 min was followed by the dilution of the resulting supernatants with the ELISA Buffer included in the kit. We performed the acetylation protocol according to the manufactor’s instruction in order to detect a [cAMP] lower than 5 pmol/mL. Finally, the samples were read at 405 nm. The concentration of cAMP was reported in terms of pmol/mg of protein. In some experiments, we performed the assay preincubating astrocytes for 24 h with pertussis toxin (PTX; 100 ng/mL; Sigma-Aldrich, Milan, Italy).

### 2.5. Immunofluorescence, Confocal Image Acquisition and Morphological Analysis

Astrocytes were plated on 12 mm pre-coated glass coverslips (5 × 10^4^ cells/coverslip). At 20–22 DIV, cells were subjected to a fixation process using paraformaldehyde (PFA) at a concentration of 4% (Sigma-Aldrich) for a duration of 15 min at RT in a solution of phosphate-buffered saline (PBS) with the following composition: 137 mM NaCl, 2.7 mM KCl, 10 mM Na_2_HPO_4_, and 1.8 mM KH_2_PO_4_. After permeabilization and saturation in PBS supplemented with Triton X-100 (0.02%; Sigma-Aldrich) and bovine serum albumin (0.5%, BSA; Sigma-Aldrich), the astrocytes were incubated overnight at 4 °C with the following primary antibodies, which were diluted in PBS containing 3% BSA: goat anti-GFAP (1:500; Santa Cruz Biotechnoloy Inc., Dallas, TX, USA); rabbit anti-GFAP (1:1000; Sigma-Aldrich); mouse anti-RIP (1:10,000, Sigma-Aldrich); mouse anti-CD11b (1:25; Sigma-Aldrich); mouse anti-Aldh1L1 (1:1000; Novus Biologicals, Bio-Techne, Milan, Italy); rabbit anti-OTR (1:100; Alomone Labs, Jerusalem, Israel); mouse anti-ezrin (1:50; Sigma-Aldrich); rabbit anti-VGLUT1 (1:400; Alomone Labs); guinea pig anti-VGLUT2 (1:200; Alomone Labs); and goat anti-EAAT2 (1:250; Santa Cruz Biotechnoloy Inc). The astrocytes were then incubated for 1 h at RT with secondary antibodies conjugated with AlexaFluor 488, 546 or 633 probes (Life Technologies Corporation, Carlsbad, CA, USA) in PBS supplemented with 3% BSA. The glass coverslips were then mounted on slides with ProLong Gold mounting medium (ThermoFisher Scientific, Waltham, MA, USA). Images were acquired by Leica STELLARIS 8 Falcon τSTED (Leica Microsystems, Mannheim, Germany) inverted confocal/stimulated emission depletion (STED) microscope. We used a white light laser to induce the excitation wavelengths, setting specific notch filters, and used three Hybrid HyD detectors. Fluorescence images were acquired using an HCX PL APO CS oil immersion objective (100×, 1.40 NA) and a 20× objective (0.70 NA), with LAS X software (version 4.8.1.29271, copyright 2025 Leica Microsystems CMS GmbH). Brightfield images were acquired using an optical inverted microscope Nikon TMS (Nikon, Amstelveen, The Netherlands). Astrocyte morphology was assessed by using the measure function in Fiji (software 2.9) [50]. GFAP stain was used to generate a cellular mask to quantify the value of cell surface area and cell perimeter. These parameters were used to determine the cell shape factor (CSF = 4 × π × S/p^2^ where S is the surface area and p the cell perimeter [51]).

### 2.6. Calculations and Statistical Analysis

The presentation of the data is the mean ± SEM of the number of experiments (*n*) indicated in each figure legend or along the text. The *t*-test, one-way ANOVA and Bonferroni’s post hoc test were performed to analyze the significance of the difference; statistical significance was set at *p* < 0.05. We used the Prism 4.02 software package (GraphPad Software, San Diego, CA, USA) to conduct the statistical analysis.

### 2.7. Materials

DMEM was purchased from Gibco while L-cysteine, BSA, PAF, 4-AP, U73122, PTX, atosiban, OT and DNAse I were from Sigma Aldrich (Milan, Italy). L 371,257 and carbetocin were purchased from Tocris (distributed in Italy Bio-Techne SRL, Milan, Italy). We used distilled water to dissolve the drugs. FBS, penicillin and streptomycin were purchased from Euroclone (Milan, Italy) while Calcium Green™-1 AM was from Life Technologies Italia (Milan, Italy). All the salt was from VWR. The used cAMP ELISA kit was from Cayman Chemical (Ann Arbor, MI, USA).

## 3. Results

### 3.1. Primary Astrocytes and OTRs Expression

**Figure 1:**
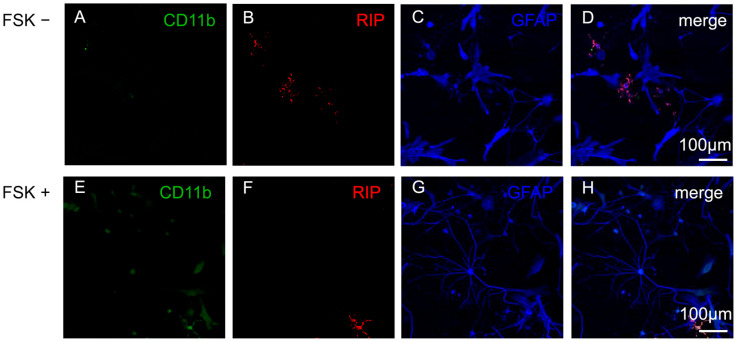
Primary astrocytes. Representative confocal images of astrocytes grown in DMEM in the absence (FSK−) or in the presence of FSK 10 µM (FSK+). Cells expressed GFAP, an astrocytic marker (**C**,**G**). Negligible contaminations from microglia (**A**,**E**) or oligodendrocytes (**B**,**F**) were detected. Merges of the single channels from the representative fields are shown in (**D**,**H**). Scale bars (100 µm) are shown. The staining was performed on three independent cell cultures, and the images are representatives of these. For additional details see Section 2.

In order to set the culture conditions, after isolation, the astrocytes were maintained in standard medium or in medium supplemented with FSK 10 µM. In both the conditions, the culture procedures, medium, shake-off and frequency of cell medium changes, were able to remove the other glial cells. We obtained highly enriched astroglial cultures with cells expressing GFAP (Figure 1C,D,G,H) and negligible contamination from microglia—detected by Integrin αM subunit CD11b (Figure 1A–H), and oligodendrocytes—detected by RIP (Receptor Interacting Protein, typical of non-myelinating oligodendrocytes progenitors, Figure 1B–D,F–H).

**Figure 2:**
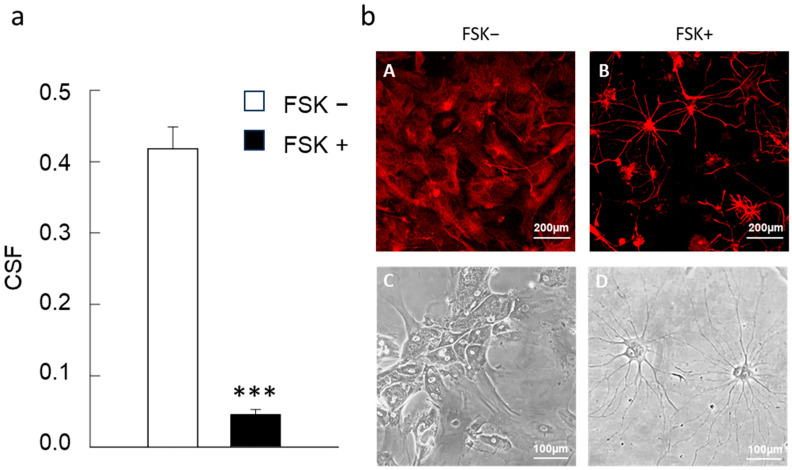
Morphological analysis. (**a**) Using GFAP immunolabeled astrocytes at 21 DIV, cell morphology was calculated in DMEM FSK− and DMEM FSK + astrocyte cultures by the cell shape factor (CSF = 4 × π × S/p^2^, where S represents the surface area and p the cell perimeter). The single z stack images were collected with confocal microscopy. Data are reported as mean ± SEM of *n* = 21 and 28 cells from three independent experiments for DMEM and FSK conditions, respectively. *** *p* < 0.001 according to a two-tail *t* Student’s test. For additional details see Section 2. (**b**) Images for primary astrocytes. (**A**,**B**) Representative confocal images using anti-GFAP primary antibody, and (**C**,**D**) representative optical microscopy images of astrocytes grown for a week in DMEM (FSK−) or in medium with 10 µM FSK added (FSK+).

Using the signaling for the astrocytic marker GFAP, the morphology of the cells was evaluated and morphometric analysis was conducted; a quantitative evaluation of the cell ramification was carried out through the “cell shape factor” as calculated by the ratio between surface area and cell perimeter [51]. Astrocytes cultured in the presence of FSK exhibited a ramified branched star-like shape, with numerous processes extending from the cell body, while astrocytes cultured in the absence of FSK appeared polygonal; the morphometric analysis confirmed the differences in astrocytes cultured in the presence of FSK (Figure 2a,b).

**Figure 3:**
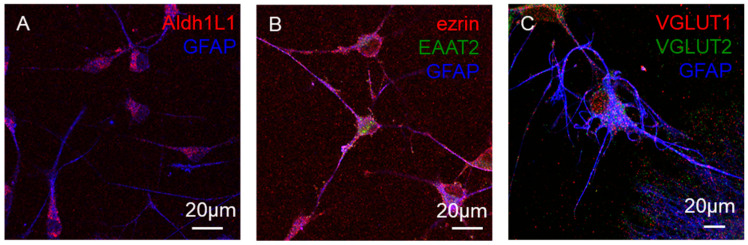
Primary astrocytes: expression of specific and functional markers. Representative confocal merged images of astrocytes grown for a week in DMEM medium with 10 µM FSK added. Images show the presence of Aldh1L1 (red, (**A**)), ezrin (red, (**B**)), EAAT2 (green, (**B**)), VGLUT1 (red, (**C**)), and VGLUT2 (green, (**C**)) on GFAP-positive astrocytes (blue, (**A**–**C**)). Scale bars (20 µm) are shown. The staining was performed on three independent cell cultures, and the images are representatives of these. Additional details are reported in Section 2.

Confocal imaging showed that primary astrocytes cultured in medium with 10 µM added FSK express, besides the GFAP astrocyte markers, the aldehyde dehydrogenase 1 family member L1 (Aldh1L1, Figure 3A) and ezrin, a preferential marker for the astrocyte processes (Figure 3B). Consistent with our images, ezrin-immunoreactive peripheral astrocyte processes were reported to cover the entire astrocytic surface in dissociated morphologically intact cortical astrocytes (see [52]). The astrocytes also express functional markers such as the excitatory amino-acid transporter type 2 EAAT2 (Figure 3B) and the vesicular glutamate transporters VGLUT1 and VGLUT2 (Figure 3C) related to the function of secreting the gliotransmitter glutamate.

Therefore, we opted for the experimental protocol involving the culture of astrocytes in the presence of FSK. All the experiments were therefore conducted on astrocytes cultured according to the DMEM FSK + protocol.

**Figure 4:**
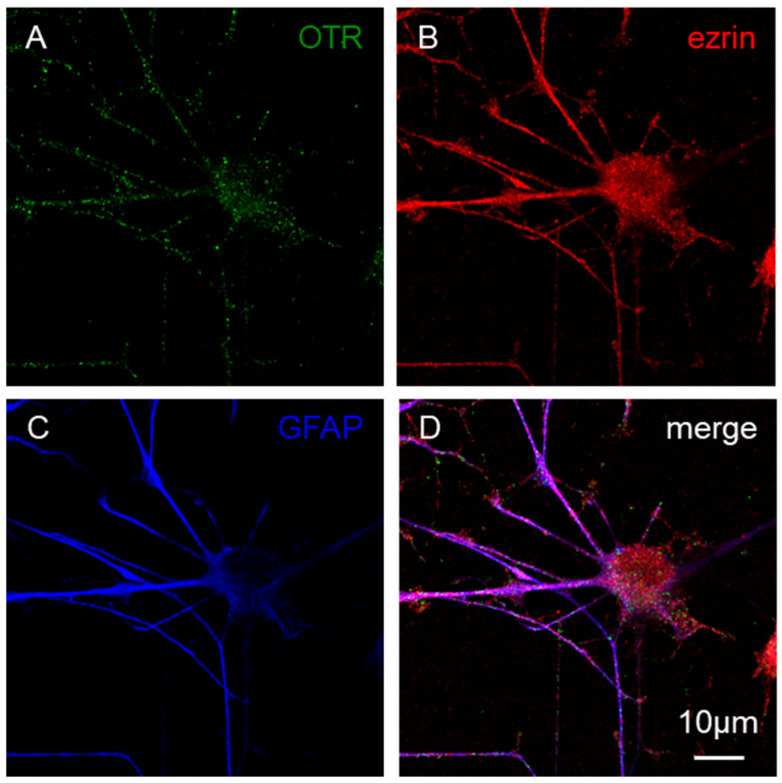
Primary astrocytes: expression of OTR. Representative confocal images of astrocytes grown for a week in DMEM medium with 10 µM FSK added. Images show the presence of OTR (green, (**A**)) on ezrin- (red, (**B**)) and GFAP-positive astrocytes (blue, (**C**)). A scale bar (10 µm) is shown in the merge (**D**). The staining was performed on three independent cell cultures, and the images are representatives of these. For additional details see Section 2.

Immunofluorescence was used to assess the expression of OTR; the receptor was expressed on ezrin- and GFAP-positive astrocytes (Figure 4) and the OTRs appeared widely distributed on both the astrocyte soma and the processes (Figure 4).

### 3.2. Ca^2+^ Signals in Astrocytes: Responses to OT and Biased OT Agonists

We previously demonstrated that OTR activation could evoke Ca^2+^ signals in resting conditions and inhibit the Ca^2+^ signals evoked by the depolarization of astrocyte processes prepared from adult striatal astrocytes [18,28].

**Figure 5:**
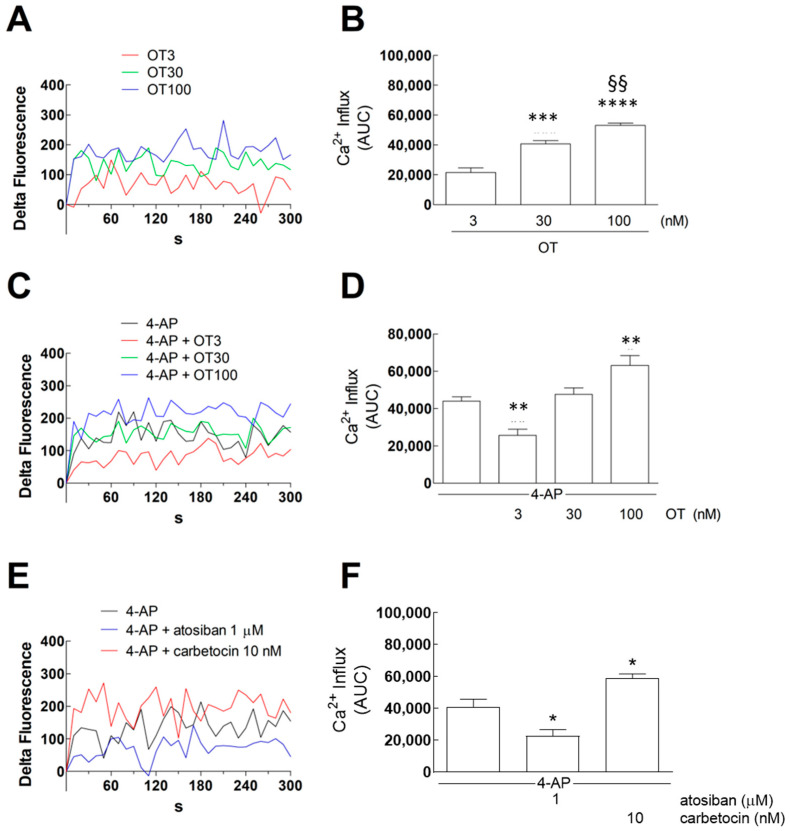
Calcium influx in response to OTR agonists and 4-AP in astrocytes. Astrocytes were pre-loaded with CG and subjected to the indicated drugs for 300 s at 37 °C. Fluorescence associated with CG was recorded every 10 s from 0 to 300 s and the measures were used to calculate the “Delta Fluorescence”, which represents the [Ca^2+^]_i_ increase over 300 s. (**A**,**C**,**E**) The graphs represent the time courses of the mean values of “Delta Fluorescence” obtained from *n* = 4–5 independent experiments. The Ca^2+^ influx after 300 s was used to calculate the Areas Under the Curves (AUCs) that are reported in (**B**,**D**,**F**) for each experimental condition. Additional details are reported in Section 2. We applied one-way ANOVA analysis to evaluate the effects of OT at the used concentrations in the basal condition ((**B**); *p* < 0.0001) and to assess the differences between the following groups: *** *p* < 0.001 or **** *p* < 0.0001 vs. OT 3 nM and §§ *p* < 0.001 compared with the effect of OT 30 nM according to Bonferroni’s post hoc test. We applied one-way ANOVA analysis to evaluate the effects of OT at the used concentrations in the presence of 4-AP 300 µM ((**D**); *p* < 0.0001) and to assess the differences between the following groups: ** *p* < 0.01 compared with the effect of 4-AP according to Bonferroni’s post hoc test. Moreover, we used one-way ANOVA analysis to evaluate the effects of the biased agonist atosiban and carbetocin in the presence of 4-AP ((**F**); *p* < 0.001) and to assess the differences between the following groups: * *p* < 0.05 compared with the effect of 4-AP according to Bonferroni’s post hoc test. 4-AP, 4-aminopyridine; CG, Calcium Green™-1 AM; OT, oxytocin.

Here, we evaluate the effects of OTR activation on the Ca^2+^ signals in FSK-treated primary astrocytes. We found that OT was effective, per se, in increasing intracellular Ca^2+^ levels in a concentration-dependent manner, with a significant increase observed at concentrations of 30 nM and 100 nM (Figure 5A,B). Then, to evaluate the effects of OT on the intracellular Ca^2+^ levels in depolarized conditions, we used 4-AP (300 µM). OT 3 nM significantly decreased the 4-AP evoked Ca^2+^ signals in astrocytes (Figure 5C,D), while at high nanomolar concentration (100 nM), it could increase the depolarization-evoked Ca^2+^ signals (Figure 5C,D).

Notably, atosiban, a biased agonist that was shown to selectively enhance OTR coupling to G_i_ [53], inhibited the 4-AP-evoked Ca^2+^ response (Figure 5E,F). In contrast, carbetocin, a biased agonist that was reported to selectively enhance OTR coupling to G_q_ [54], augmented the 4-AP-evoked Ca^2+^ response (Figure 5E,F). The concentrations we used for carbetocin (10 nM) and atosiban (1 μM) are 100-fold different, yet they likely reflect different receptor binding affinity. Indeed, the biased agonist concentrations—which we found effective on glutamate release and Ca^2+^ signals in isolated processes from adult astrocytes [30]—were selected based on previous observations by Chini’s group: the calculated atosiban half maximal effective concentration EC_50_ on G_αi3_ subunit was 2800 ± 1035 nM [35]; 10 nM corresponded to the carbetocin affinity for OTR [54]. It has to be noted that the astrocytic Ca^2+^ signal lasted several minutes; although we did not directly measure the ion concentration but quantified the Ca^2+^ response as a difference in fluorescence, we cannot exclude that the evoked Ca^2+^ signal might potentially contribute to excitotoxicity in bulk experiments or in turn further induce glutamate release.

### 3.3. Release of Glutamate from Astrocytes: Responses to OT and Biased OT Agonists

**Figure 6:**
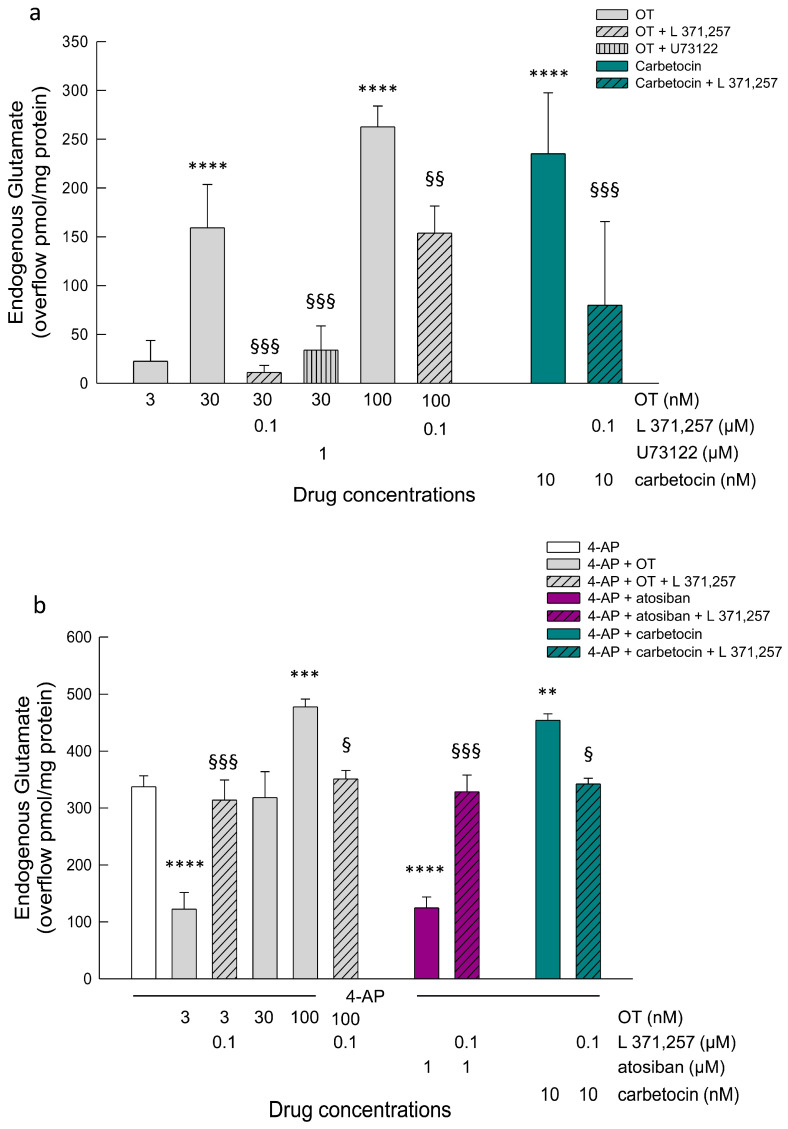
Efflux of endogenous glutamate in response to activation of OTR in astrocytes. (**a**) Glutamate release in the basal condition: lack of effectiveness of OT at 3 nM and increase in the glutamate efflux by OT at 30 nM and 100 nM, and by the biased agonist carbetocin 10 nM. Antagonism of the release evoked by OT and carbetocin by the selective OTR antagonist L 371,257. Modulation of the OT 30 nM evoked release by the phospholipase PLC inhibitor U73122. The graph shows the overflow of the endogenous glutamate presented as pmol/mg of protein in the presence of the drugs at the concentrations indicated. OT was applied for 6 min during superfusion; L 371,257 or U73122 were added to the medium 8 min before OT. For more information about the experiments, see Section 2 entitled ‘Materials and Methods’. Data are expressed as mean ± SEM of *n* = 5–7 independent experiments. We applied one-way ANOVA analysis to evaluate the effects of OT at different concentrations in the basal condition (*p* < 0.0001) and to assess the differences between the following groups: **** *p* < 0.0001 vs. OT 3 nM; §§§ *p* = 0.0001 and §§ *p* <0.001 compared with the effect of OT or cabetocin at the same concentration but in the absence of L 371,257 or U73122 according to Bonferroni’s post hoc test. OT, oxytocin. (**b**) Glutamate release in the 4-AP (300 µM) condition: inhibition of the glutamate efflux by OT 3 nM, ineffectiveness of OT 30 nM and increase by OT 100 nM. While the biased agonist atosiban mimicked the OT 3 nM, the biased agonist carbetocin 10 nM increased the 4-AP evoked glutamate release. Antagonism by the selective OTR antagonist L 371,257. The graph shows the overflow of the endogenous glutamate expressed as pmol/mg of protein in the presence of the drugs at the concentrations indicated. During superfusion, 4-AP, OT, atosiban or carbetocin were applied for 6 min; L 371,257 was added 8 min before the agonists. For more information about the experiments, see Section 2 entitled ‘Materials and Methods’. Data are expressed as mean ± SEM of *n* = 3–11 independent experiments. We applied one-way ANOVA analysis to evaluate the effects of OT at different concentrations, atosiban or carbetocin in the 4-AP condition (*p* < 0.0001) and to assess the differences between the following groups: **** *p* < 0.0001, *** *p* < 0.001 and ** *p* < 0.01 compared with the effect of 4-AP; and §§§ *p* < 0.001 and § *p* < 0.05 compared with the effect of agonist OT, atosiban or carbetocin at the same concentration in the absence of L 371,257 according to Bonferroni’s post hoc test. OT, oxytocin. 4-AP, 4-aminopyridine.

We investigated on the release of endogenous glutamate from FSK-treated primary astrocytes during superfusion. The first two fractions collected showed that the superfusion of astrocytes with the standard medium resulted in a glutamate outflow of 674.7 ± 61.51 pmol/mg protein in the 3 min sample (*n* = 13). Exposure to 4-AP (300 µM) resulted in increased the glutamate efflux (Figure 6).

We previously reported that in astrocyte processes prepared from adult striatal astrocytes [18,28,30], OT in the nanomolar range could evoke the release of the gliotransmitter glutamate in resting conditions and inhibit the efflux evoked by depolarization.

Here, we observed that OT 3 nM did not affect the basal glutamate efflux from primary astrocytes, while at 30 nM and 100 nM, OT could evoke an endogenous glutamate response from the astrocytes (Figure 6a). The overflow evoked by OT 30 nM was prevented, and that evoked by OT 100 nM was significantly reduced in the presence of the OTR antagonist L 371,257, indicating that the responses involved OTR activation (Figure 6a). Furthermore, the PLC inhibitor U73122 could inhibit the response to OT 30 nM, indicating the involvement of the G_q_-PLC pathway in the glutamate releasing response (Figure 6a). The biased agonist carbetocin selectively promoting OTR coupling to G_q_ [54] evoked a glutamate overflow that was inhibited by L 371,257, indicating the involvement of OTR in the response (Figure 6a). The compounds L 371,257 and U73122 did not affect the glutamate outflow.

Moreover, OT 3 nM was found to inhibit the 4-AP evoked glutamate release from astrocytes, while OT 30 nM could not modulate the 4-AP-evoked release of glutamate (Figure 6b). The biased agonist atosiban, selectively promoting OTR coupling to G_i_ [53], inhibited the 4-AP-evoked glutamate (Figure 6b). Conversely, the biased agonist carbetocin increased the glutamate efflux evoked by 4-AP (Figure 6b). The inhibitory effect of OT 3 nM or atosiban, and the facilitatory effect of OT 100 nM or carbetocin on the 4-AP-evoked glutamate efflux were significantly reduced by the OTR antagonist L 371,257, indicating that the responses involved OTR activation (Figure 6b).

### 3.4. cAMP Production in Astrocytes: Responses to OT and the Biased OT Agonist Atosiban

**Figure 7:**
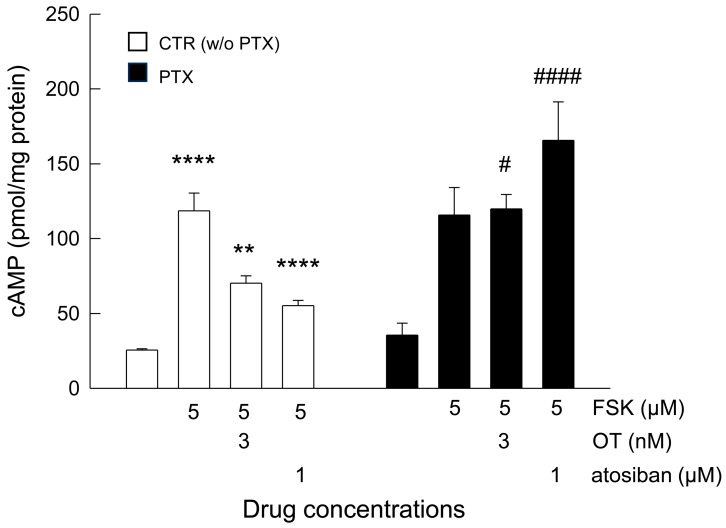
cAMP production and modulation in astrocytes by OTR activation. ELISA showed the effect of OT and atosiban on the FSK-evoked cAMP production. Astrocytes were used at 20–22 DIV. The day before the experiment, the cell medium was replaced with DMEM without supplements. To increase cAMP levels, FSK (5 µM) was applied for 15 min in the absence or in the presence of OT or atosiban at the shown concentrations. PTX (100 ng/mL) was added to the cell medium 24 h before the experiments. For each experiment, at least one well was conducted in simple medium and used as a control (CTR). The amount of cAMP was expressed as pmol/mg protein. Data are expressed as mean ± SEM of *n* = 4–9 independent experiments. We applied one-way ANOVA to evaluate the effects of OT and atosiban (*p* < 0.0001), and the Bonferroni post hoc test was used for multiple comparison analysis between the groups; the adjusted p values are reported here: **** *p* < 0.0001 CTR vs. FSK and FSK vs. FSK + atosiban; ** *p* = 0.0024 FSK vs. FSK + OT 3 nM; # *p* = 0.0113 FSK + OT 3 nM vs. FSK + OT 3 nM in PTX; #### *p* < 0.0001 FSK + atosiban vs. FSK + atosiban in PTX; ns CTR vs. CTR in PTX, FSK vs. FSK in PTX, and FSK + atosiban in PTX vs. FSK in PTX. OT, oxytocin; FSK, forskolin; PTX, pertussis toxin.

In the attempt to understand the inhibitory effect of OT in astrocytes, and bearing in mind that in neuronal cells, OTR has also been linked to an inhibitory pathway, we explored this possibility at molecular level. Therefore, we assessed whether OTR coupling to G_i_ with subsequent inhibition of adenylyl cyclase and cAMP signaling [35,55,56,57] could be involved in the responses to OT in astrocytes. After leaving the astrocytes in simple DMEM without any supplements, they were acutely stimulated with FSK, a direct activator of adenylyl cyclase capable of causing significant increases in intracellular [cAMP] [58]. Under these conditions, we then evaluated the effect of OT and atosiban on the cAMP response evoked by FSK. FSK could increase intracellular cAMP (Figure 7); the cAMP increase was inhibited by OT 3 nM and by atosiban 1 µM, the biased agonist reported to selectively promote OTR coupling to G_i_ [53] (Figure 7). To assess the involvement of the G_i/o_ pathway—a PTX-sensitive mechanism [55,59], as PTX acts to uncouple G_i/o_ proteins by ADP-ribosylating these subunits [60])—we checked the effect of PTX on the inhibitory effect. PTX did not change the resting cAMP levels, nor the FSK-evoked cAMP production (Figure 7). On the other hand, the inhibitory effect of OT or atosiban on the FSK-evoked cAMP production was prevented by PTX pretreatment (Figure 7), indicating the involvement of PTX-sensitive-G_i/o_ proteins in the OT and atosiban effects.

**Figure 8:**
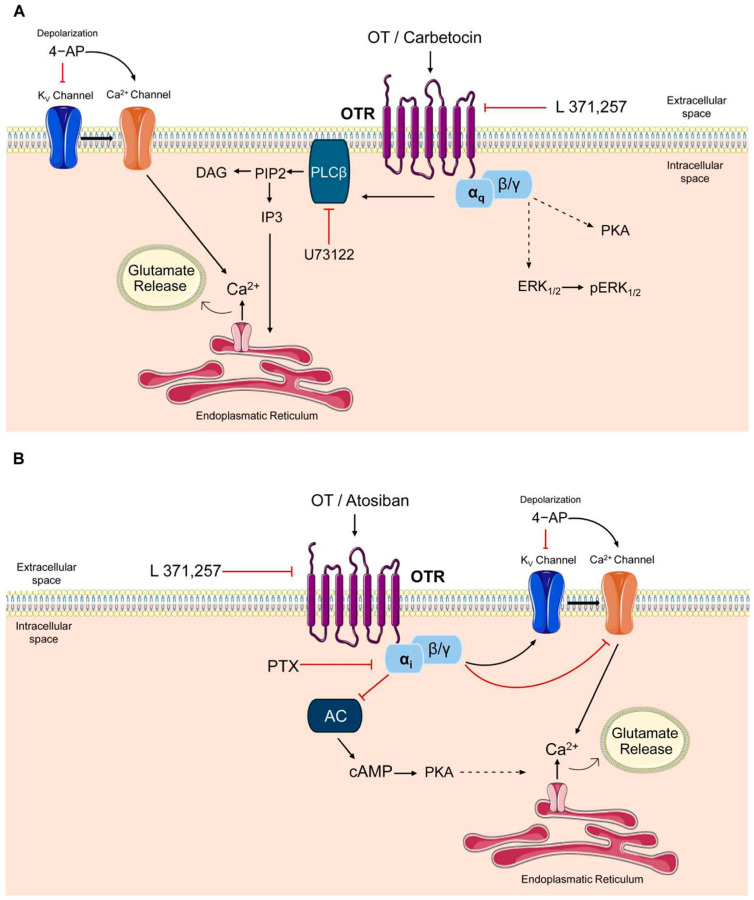
Overview of OTR molecular pathways: effects of OT and biased agonists. (**A**) Oxytocin and the biased agonist carbetocin induce OTR coupling to G_q_ protein. G_αq_ promotes phospholipase Cβ (PLCβ) activation that metabolizes phosphatidylinositol-4,5-bisphosphate (PIP2) into diacylglycerol (DAG) and inositol 1,4,5-triphosphate (IP3). The production of IP3 facilitates the mobilization of calcium (Ca^2+^) from the intracellular stores, leading to glutamate release. U73122 inhibits PLCβ activation. In astrocytes, the OT-pathway through OTR coupling to G_αq_ also induces G_βγ_-mediated activation of ERK (extracellular signal-regulated kinases) and PKA (protein kinase A) [61]. (**B**) Oxytocin and the biased agonist atosiban induce OTR coupling to G_i_ protein, which inhibits the enzyme adenylyl cyclase (AC), thereby reducing the production of the second messenger cyclic adenosine monophosphate (cAMP). Pertussis toxin (PTX) blocks the α subunit of the G_i_ protein in an inactive state, decreasing cAMP production. On the other hand, the OT-pathway through OTR coupling to G_αi_ also induces G_βγ_-mediated modulation of K^+^ and Ca^2+^ channels [62]. The black arrows indicate an activating/stimulating effect. The red lines, on the other hand, indicate an inhibitory effect. 4−aminopyridine (4-AP) is a blocker of K_v_ channels but could also directly facilitate Ca^2+^ entry through voltage-gated channels [45]. L 371,257 is a competitive OTR antagonist.

These findings substantiate the hypothesis that OTR can couple to a PTX-sensitive-G_i_-mediated pathway responsible for the inhibition of cAMP signal in stimulated conditions. A summarizing schematic diagram of the mechanisms of action of the agonists, of the antagonist and of the inhibitor is shown in Figure 8.

## 4. Discussion

The evidence reported here was obtained in primary cultures of astrocytes. Here, we report that astrocytes cultured in the presence of FSK acquired morphological and functional features of mature-like astrocytes and could respond with Ca^2+^ signals and the release of endogenous glutamate to 4-AP depolarization; primary astrocytes expressed OTR; at nanomolar concentrations, OT induced dual responses, namely the facilitation and inhibition of both Ca^2+^ signals and glutamate release; the excitatory and the inhibitory effects of OT were dependent on the activation of the G_q_ and G_i_ pathway, respectively; and the OT facilitatory and inhibitory effects were duplicated by the biased agonists carbetocin and atosiban, respectively.

### 4.1. Astrocyte Primary Culture: Morphological Analysis

Astrocytes cultured in control medium or in medium with added FSK both expressed the astrocyte marker GFAP with negligible contamination by microglia or oligodendrocytes. However, only astrocytes cultured in the presence of FSK exhibited morphological features of mature-like astrocytes with a ramified branched star-like shape, as also confirmed by the morphometric analysis with the “cell shape factor” [51]. Furthermore, they express markers related to the function of releasing the gliotransmitter glutamate and regulating glutamate extracellular levels and glutamatergic transmission. In fact, they expressed VGLUT1 and VGLUT2, which were reported to be expressed by mature freshly isolated astrocytes (see [63]) and the excitatory amino-acid transporter EAAT2, which is typically expressed by astrocytes [64]. Astrocytes cultured in medium with added FSK were then used as the experimental model in our study, and immunofluorescence and functional data were obtained from the model.

### 4.2. Astrocytes Express OTRs

Astrocytes expressed OTRs on GFAP and ezrin-positive structures; the receptors were widely distributed in the soma as well as in the branches of astrocytes, including the fine processes. Notably, OTR expression was described in cortical astrocytes in human postmortem frontal samples [20], while in rodents, astrocytic OTR expression was reported in several brain regions including the hippocampus, hypothalamus, amygdala, and auditory cortex (see [61]). We previously reported the presence of OTR on adult rat striatal astrocytes and on their processes [18,28,29,30]. As previously mentioned, primary rodent hypothalamic or spinal astrocytes were also described to express OTRs [21,22,61] on both the soma and the processes.

### 4.3. OT Induces Dual Responses in Astrocytes, Namely Facilitation and Inhibition of Both the Ca^2+^ Signals and Glutamate Release

The OTR activation in astrocytes evoked dual responses, namely the stimulation and inhibition of Ca^2+^ signals and of the release of the gliotransmitter glutamate. It is noteworthy that the OT effects on glutamate release duplicated the effects on Ca^2+^ signals, suggesting that OT might regulate Ca^2+^-dependent release of glutamate; indeed, the presence of VGLUTs is consistent with Ca^2+^-dependent vesicular glutamate release from primary astrocytes. In fact, although for decades it was believed that vesicular mechanisms of transmitter release were missed in astrocytes, in the last 40 years, evidence was provided that astrocytes are equipped with mechanisms for vesicular gliotransmitter release, and that in response to an increase in intracellular Ca^2+^, the vesicular membrane can fuse with the plasma membrane, letting gliotransmitters exit into the extracellular space [65,66]. Consistently, here we find that astrocytes are equipped with vesicular glutamate transporters to load the vesicles with the gliotransmitter glutamate. We then propose that the intracellular Ca^2+^ increase due to 4-AP depolarization or activation of G_q_ protein-coupled receptors is likely to activate fusion of the vesicle membrane with the plasma membrane, allowing vesicular glutamate release. Indeed, 4-AP by blocking voltage-gated K^+^ channels and mimicking the physiological mechanisms of membrane depolarization is considered a quasi-physiological stimulus to produce a Ca^2+^-dependent release of transmitters ([67,68] and references therein). OT could evoke Ca^2+^ signals and glutamate efflux in resting conditions and facilitate the Ca^2+^ signal and the glutamate efflux in response to 4-AP depolarization. Notably, the finding in cultured astrocytes replicated the finding in the processes prepared from adult striatal astrocytes, where OTR activation could stimulate both resting and 4-AP-evoked Ca^2+^ signals and glutamate release [18,28,29,30]. Conversely, OT inhibition of Ca^2+^ signals and glutamate release in primary astrocytes was observed when the membrane was depolarized by 4-AP, replicating the finding in the processes prepared from adult striatal astrocytes (see [18,28,29,30]). Nevertheless, we must consider that primary astrocytes are a simplified model: they reproduce astrocytic responses to depolarization, not the full complexity of neuronal firing patterns and synaptic signaling. The ability of OT to regulate, either facilitating or inhibiting, Ca^2+^ events and the release of glutamate in astrocytes, deserves a comment. The mechanism by which OT facilitates or inhibits Ca^2+^ signal may be related to the canonical pathways dependent on G_q_-coupled receptors or G_i_-coupled receptors, respectively. Facilitation may be related to the activation of G_q_ and then to inositol 1,4,5-trisphosphate-mediated Ca^2+^ release from internal stores [69]. Inhibition may be related to recruitment of the G_αi/o_ protein, which inhibits adenylyl cyclase and thus reduces intracellular cAMP, while the G_βγ_ subunit activates inwardly rectifying K^+^ channels and/or inhibits Ca^2+^ channels [62]. Decreased Ca^2+^ levels can be presumed to be the resulting action of the G_βγ_ subunit, as already reported in astrocytes (see [70]). The finding is of interest when considering that only excitatory Ca^2+^ signals were reported following OTR activation in astrocytes [26,27]. At variance, both inhibitory and excitatory effects of OT [71] were described in neuronal cells ([72,73,74]; also see below). Furthermore, it is worth noting that in primary astrocytes, the effect of OT could be both inhibitory and excitatory, or only inhibitory, depending on the membrane depolarization; thus, the membrane state of the astrocytes appears to drive the response to OT. In fact, astrocyte depolarization has been reported in response to neuronal activity [6,75,76,77,78].

Finally, both the excitatory and inhibitory effects were evoked by nanomolar OT, coherent with effective OT concentrations at OTR in the brain (see [79]). This is in contrast with what was reported with transfected OTR, where G_q_ signaling could be activated by OT with an EC_50_ of about 2 nM, whereas G_i/o_ isoforms required EC_50_ values ranging from 10 nM to 100 nM (see [79]). In native OTR, here we obtain evidence for an inhibitory effect (on depolarized astrocytes) at a low nanomolar concentration (3 nM), while excitatory effects appeared at higher concentrations (30–100 nM). The finding is consistent with our previous findings on native OTR in processes of mature astrocytes, where 3 nM OT inhibited the evoked glutamate release and Ca^2+^ signal, while at 30 nM, it facilitated it.

### 4.4. The Excitatory and Inhibitory Effects of OT Were Dependent on Activation of the G_q_ and G_i_ Pathway, Respectively

It was described that OTR can couple to both G_q_-dependent and G_i_-dependent pathways [36]. In neuronal cells coupling OTRs to G_q_ and PLC, inositol-1,4,5-triphosphate signaling was described to evoke Ca^2+^ signals and neuron excitation (see [57,71,80]), while presynaptic OTRs coupling to G_i_ modulated the Ca^2+^ entry into the nerve terminals and inhibited neurotransmitter release [81,82,83]. Analogously, we recently proposed that in astrocyte processes, G_q_ signaling could mediate excitatory OT actions with increased intracellular Ca^2+^ level and glutamate release, while coupling to G_i_ pathways could modulate Ca^2+^ entry and gliotransmitter release (see [30]). By investigating the molecular mechanisms of the receptor transduction, here we find that the PLC blocker U73122 abolished OT’s ability to evoke glutamate release from astrocytes, indicating the involvement of the G_q_-PLC pathway in the OT excitatory effect. The finding is in line with the previously reported ability of OT to activate excitatory G_q_-dependent pathways in primary astrocytes [26,84]. Conversely, OT inhibited the cAMP level increased by direct adenylyl cyclase activation by FSK, supporting the idea that the OT inhibitory effect was dependent on coupling to a G_i_ pathway and inhibition of adenylyl cyclase. The finding is in accordance with the recently reported OT-dependent G_i_ activation in cultured hypothalamic astrocytes [84]. PTX, inhibiting the responses mediated by the G_i/o_ pathway [59], could abolish the OT inhibition of the evoked cAMP production, demonstrating the involvement of PTX-sensitive G_i/o_ proteins. Therefore, we can assume that when astrocytic membrane is depolarized and therefore cAMP synthesis is activated [85], inhibitory OT effects mediated by OTR coupling to the G_i/o_ pathway can emerge.

### 4.5. The OT Facilitatory and Inhibitory Effects Were Duplicated by the Biased Agonists Carbetocin and Atosiban, Respectively

Carbetocin, an OT-derived substance which selectively engages the G_q_ pathway [54], mimicked the stimulatory OT effect on depolarization-evoked Ca^2+^ signals and on the resting or 4-AP-evoked glutamate efflux. Atosiban, a biased ligand for OTR displaying agonistic activity only at the G_i_ pathway [53,86], mimicked the inhibitory OT effects on both depolarization-evoked Ca^2+^ signals and glutamate efflux, and on the cAMP response to FSK in a PTX-sensitive way. Notably, at variance with what was reported in slices or in in situ astrocytes where G_i_ protein-coupled receptors could evoke astrocytic Ca^2+^ transients (see [24,87]), only inhibition of Ca^2+^ signal (or glutamate release) was induced by the biased agonist atosiban and no Ca^2+^ transient could be observed in response to G_i_ activation.

Our findings are consistent with the dual OT effect and support the ability of OTR to couple to both G_q_ and G_i/o_ pathways in primary astrocytes to regulate intracellular Ca^2+^ signals and the efflux of glutamate. Furthermore, the findings on the biased agonists validate the model of primary astrocytes for astrocytic OTR functional studies, confirming that they express OTRs capable of coupling to dual pathways. In fact, the finding in cultured astrocytes can reproduce the finding in astrocyte processes prepared form adult astrocytes that have matured in astrocyte–neuron networks (see [30]).

As a limitation of the study, we must consider that cultured astrocytes may not reflect in vivo conditions, and any extrapolation to astrocyte functioning in astrocyte–neuron networks would be speculative. Furthermore, the interpretation of dual G_q_/G_i_ pathway coupling may oversimplify the G protein-coupled receptor signaling and mechanisms of glutamate release, although the effects of U73122 and PTX indicate that the G_q_-PLC pathway and a PTX-sensitive G_i/o_ pathway are involved in the glutamate release responses to OT. Another limitation may be related to the OT concentration. Indeed, although nanomolar concentrations are considered physiological (see [79]), OT is a neuropeptide mainly acting through volume transmission, so that its concentration in the receptor biophase is expected to vary in time depending on the brain region, the source and time of secretion and on the receptor localization. In fact, OT effects in a wide range of concentrations, including very low nanomolar or sub-nanomolar concentrations [84], might be of physiological significance.

## 5. Conclusions

In conclusion, here we provide evidence showing that OT is able to regulate Ca^2+^ signals and the release of the gliotransmitter glutamate in rodent primary astrocytes. In fact, in response to OT in the nanomolar range, we could observe not only an increased Ca^2+^ signal and glutamate release, but also the inhibition of both the Ca^2+^ signal and glutamate release. Investigation on molecular signaling indicated that OT was able to activate both G_q_-PLC-excitatory and G_i_-dependent pathways in astrocytes, depending on the membrane depolarization. While in primary astrocytic cultures, OT was reported to evoke excitatory responses and Ca^2+^ signals, to our knowledge, this is the first report of OT’s inhibitory effects on Ca^2+^ signals and glutamate release in astrocytes.

Notably, in primary astrocyte cultures, here we confirm the findings in astrocyte processes freshly prepared from astrocytes matured in a neuron–astrocyte network: in fact, both excitatory and inhibitory effects of OT were detected in astrocyte processes prepared from adult striatal astrocytes. We can conclude that our experimental protocol, involving culturing astrocytes in a medium with added FSK, allows the astrocytes to exhibit not only morphological features, but also functional responses (depolarization-evoked release and Ca^2+^ signals) similar to those exhibited by adult astrocytes. We therefore propose that primary astrocyte cultures treated with FSK may represent a suitable model to study astrocyte features and to investigate astrocyte functioning and response to molecular signals.

## Data Availability

The dataset is available on request from the authors; the raw data supporting the conclusions of this article will be made available by the authors on request. In fact, the data presented in this article are the basis for ongoing studies also related to the PhD thesis by Elisa Farsetti. Requests to access the datasets should be directed to C. Cervetto.

## Abbreviations

The following abbreviations are used in this manuscript

4-AP	4-aminopyridine
Aldh1L1	aldehyde dehydrogenase 1 family member L1
AUC	area under the curve
cAMP	cyclic adenosine monophosphate
BSA	bovine serum albumin
CG	calcium green
CSF	cell shape factor
DIV	days in vitro
DMEM	Dulbecco’s Modified Eagle Medium
EAAT2	excitatory amino-acid transporter type 2
EC_50_	half maximal effective concentration
ELISA	enzyme-linked immunosorbent assay
ERK	extracellular signal-regulated kinases
FBS	fetal bovine serum
FSK	forskolin
GFAP	glial fibrillary acidic protein
HPLC	high-performance liquid chromatography
IP3	inositol-1,4,5-triphosphate
OT	oxytocin
OTR	oxytocin receptor
PBS	phosphate-buffered saline
PFA	paraformaldehyde
PIP2	phosphatidylinositol-4,5-bisphosphate
PLC	phospholipase C
PKA	protein kinase A
PTX	pertussis toxin
RIP	receptor-interacting protein
RT	room temperature
STED microscopy	stimulated emission depletion microscopy
VGLUT	vesicular glutamate transporter

## References

[1] Russell J.A., Brunton P.J. (2017). Oxytocin: Control of Secretion by the Brain and Central Roles. Reference Module in Neuroscience an Biobehavioral Psychology.

[2] Froemke R.C., Young L.J. (2021). Oxytocin, Neural Plasticity, and Social Behavior. Annu. Rev. Neurosci..

[3] Mitre M., Minder J., Morina E.X., Chao M.V., Froemke R.C. (2018). Oxytocin Modulation of Neural Circuits. Curr. Top. Behav. Neurosci..

[4] Talpo F., Spaiardi P., Castagno A.N., Maniezzi C., Raffin F., Terribile G., Sancini G., Pisani A., Biella G.R. (2023). Neuromodulatory functions exerted by oxytocin on different populations of hippocampal neurons in rodents. Front. Cell. Neurosci..

[5] Castagno A.N., Spaiardi P., Trucco A., Maniezzi C., Raffin F., Mancini M., Nicois A., Cazzola J., Pedrinazzi M., Debal Papa P. (2024). Oxytocin Modifies the Excitability and the Action Potential Shape of the Hippocampal CA1 GABAergic Interneurons. Int. J. Mol. Sci..

[6] Schipke C.G., Kettenmann H. (2004). Astrocyte responses to neuronal activity. Glia.

[7] Armbruster M., Naskar S., Garcia J.P., Sommer M., Kim E., Adam Y., Haydon P.G., Boyden E.S., Cohen A.E., Dulla C.G. (2022). Neuronal activity drives pathway-specific depolarization of peripheral astrocyte processes. Nat. Neurosci..

[8] Porter J.T., McCarthy K.D. (1997). Astrocytic neurotransmitter receptors in situ and in vivo. Prog. Neurobiol..

[9] Perea G., Navarrete M., Araque A. (2009). Tripartite synapses: Astrocytes process and control synaptic information. Trends Neurosci..

[10] Nedergaard M., Verkhratsky A. (2012). Artifact versus reality-how astrocytes contribute to synaptic events. Glia.

[11] Li X., Ding L., Nie H., Deng D.Y.B. (2025). Calcium Signaling in Astrocytes and Its Role in the Central Nervous System Injury. Mol. Neurobiol..

[12] Haydon P.G., Carmignoto G. (2006). Astrocyte control of synaptic transmission and neurovascular coupling. Physiol. Rev..

[13] Rossi D., Volterra A. (2009). Astrocytic dysfunction: Insights on the role in neurodegeneration. Brain Res. Bull..

[14] Brandebura A.N., Paumier A., Onur T.S., Allen N.J. (2023). Astrocyte contribution to dysfunction, risk and progression in neurodegenerative disorders. Nat. Rev. Neurosci..

[15] Wang Y.F., Hatton G.I. (2006). Mechanisms underlying oxytocin-induced excitation of supraoptic neurons: Prostaglandin mediation of actin polymerization. J. Neurophysiol..

[16] Mitre M., Marlin B.J., Schiavo J.K., Morina E., Norden S.E., Hackett T.A., Aoki C.J., Chao M.V., Froemke R.C. (2016). A distributed network for social cognition enriched for oxytocin receptors. J. Neurosci..

[17] Wahis J., Baudon A., Althammer F., Kerspern D., Goyon S., Hagiwara D., Lefevre A., Barteczko L., Boury-Jamot B., Bellanger B. (2021). Astrocytes mediate the effect of oxytocin in the central amygdala on neuronal activity and affective states in rodents. Nat. Neurosci..

[18] Amato S., Averna M., Guidolin D., Pedrazzi M., Pelassa S., Capraro M., Passalacqua M., Bozzo M., Gatta E., Anderlini D. (2022). Heterodimer of A2A and oxytocin receptors regulating glutamate release in adult striatal astrocytes. Int. J. Mol. Sci..

[19] Althammer F., Roy R.K., Lefevre A., Najjar R.S., Schoenig K., Bartsch D., Eliava M., Feresin R., Hammock E.A.D., Murphy A.Z. (2022). Altered PVN-to-CA2 hippocampal oxytocin pathway and reduced number of oxytocin-receptor expressing astrocytes in heart failure rats. J. Neuroendocr..

[20] McKay E.C., Beck J.S., Khoo S.K., Dykema K.J., Cottingham S.L., Winn M.E., Paulson H.L., Lieberman A.P., Counts S.E. (2019). Peri-infarct upregulation of the oxytocin receptor in vascular dementia. J. Neuropathol. Exp. Neurol..

[21] Di Scala-Guenot D., Strosser M.T. (1992). Oxytocin receptors on cultured astroglial cells. Kinetic and pharmacological characterization of oxytocin-binding sites on intact hypothalamic and hippocampic cells from foetal rat brain. Biochem. J..

[22] Evrard M.E., Strosser M.T., Di Scala-Guenot D. (1997). Pharmacological characterization of oxytocin-binding sites in rat spinal cord membranes: Comparison with embryonic cultured spinal cord neurones and astrocytes. J. Neuroendocr..

[23] Meinung C.P., Boi L., Pandamooz S., Mazaud D., Ghézali G., Rouach N., Neumann I.D. (2025). OXTR-mediated signaling in astrocytes contributes to anxiolysis. Mol. Psychiatry.

[24] Baudon A., Grelot V., Wang K.Y., Althammer F., Denis C., Riché-Piotaix P., Ali A.S., Yan Y., Castillo Díaz F., Piacentini F. (2025). Stress induces oxytocin-Gαi-dependent remodeling of astrocytes to shape neuronal response in the amygdala. Nat. Commun..

[25] Langle S.L., Poulain D.A., Theodosis D.T. (2003). Induction of rapid, activity-dependent neuronal-glial remodelling in the adult rat hypothalamus in vitro. Eur. J. Neurosci..

[26] Di Scala-Guenot D., Mouginot D., Strosser M.T. (1994). Increase of intracellular calcium induced by oxytocin in hypothalamic cultured astrocytes. Glia.

[27] Kuo J., Hariri O.R., Micevych P. (2009). An interaction of oxytocin receptors with metabotropic glutamate receptors in hypothalamic astrocytes. J. Neuroendocr..

[28] Amato S., Averna M., Guidolin D., Ceccoli C., Gatta E., Candiani S., Pedrazzi M., Capraro M., Maura G., Agnati L.F. (2023). Heteromerization of Dopamine D2 and Oxytocin Receptor in Adult Striatal Astrocytes. Int. J. Mol. Sci..

[29] Amato S., Averna M., Farsetti E., Guidolin D., Pedrazzi M., Gatta E., Candiani S., Maura G., Agnati L.F., Cervetto C. (2024). Control of Dopamine Signal in High-Order Receptor Complex on Striatal Astrocytes. Int. J. Mol. Sci..

[30] Farsetti E., Amato S., Averna M., Guidolin D., Pedrazzi M., Maura G., Agnati L.F., Cervetto C., Marcoli M. (2025). Dual oxytocin signal in striatal astrocytes. Biomolecules.

[31] Ferroni S., Marchini C., Schubert P., Rapisarda C. (1995). Two distinct inwardly rectifying conductances are expressed in long term dibutyryl-cyclic-AMP treated rat cultured cortical astrocytes. FEBS Lett..

[32] McManus M.F., Chen L.C., Vallejo I., Vallejo M. (1999). Astroglial differentiation of cortical precursor cells triggered by activation of the cAMP-dependent signaling pathway. J. Neurosci..

[33] Paco S., Margelí M.A., Olkkonen V.M., Imai A., Blasi J., Fischer-Colbrie R., Aguado F. (2009). Regulation of exocytotic protein expression and Ca^2+^-dependent peptide secretion in astrocytes. J. Neurochem..

[34] Paco S., Hummel M., Plá V., Sumoy L., Aguado F. (2016). Cyclic AMP signaling restricts activation and promotes maturation and antioxidant defenses in astrocytes. BMC Genom..

[35] Busnelli M., Saulière A., Manning M., Bouvier M., Galés C., Chini B. (2012). Functional Selective Oxyto-cin-derived Agonists Discriminate between Individual G Protein Family Subtypes. J. Biol. Chem..

[36] Jurek B., Neumann I.D. (2018). The Oxytocin Receptor: From Intracellular Signaling to Behavior. Physiol. Rev..

[37] McCarthy K.D., de Vellis J. (1980). Preparation of separate astroglial and oligodendroglial cell cultures from rat cerebral tissue. J. Cell Biol..

[38] Gottfried C., Cechin S.R., Gonzalez M.A., Vaccaro T.S., Rodnight R. (2003). The Influence of the Extracellular Matrix on the Morphology and Intracellular PH of Cultured Astrocytes Exposed to Media Lacking Bicarbonate. Neuroscience.

[39] Won C.L., Oh Y.S. (2000). cAMP-induced stellation in primary astrocyte cultures with regional heterogeneity. Brain Res..

[40] Favero C.B., Mandell J.W. (2007). A pharmacological activator of AMP-activated protein kinase (AMPK) induces astrocyte stellation. Brain Res..

[41] Saura J. (2007). Microglial cells in astroglial cultures: A cautionary note. J. Neuroinflamm..

[42] Verkhratsky A., Nedergaard M. (2018). Physiology of Astroglia. Physiol. Rev..

[43] Bordey A., Sontheimer H. (1999). Differential inhibition of glial K^+^ currents by 4-AP. J. Neurophysiol..

[44] Verkhratsky A., Steinhäuser C. (2000). Ion channels in glial cells. Brain Res. Rev..

[45] Rogawski M.A., Barker J.L. (1983). Effects of 4-aminopyridine on calcium action potentials and calcium current under voltage clamp in spinal neurons. Brain Res..

[46] Barres B.A., Chun L.L., Corey D.P. (1989). Calcium current in cortical astrocytes: Induction by cAMP and neurotransmitters and permissive effect of serum factors. J. Neurosci..

[47] Bradford M.M. (1976). A rapid and sensitive method for the quantitation of microgram quantities of protein utilizing the principle of protein-dye binding. Anal. Biochem..

[48] Benfenati V., Caprini M., Nicchia G.P., Rossi A., Dovizio M., Cervetto C., Nobile M., Ferroni S. (2009). Carbenoxolone inhibits volume-regulated anion conductance in cultured rat cortical astroglia. Channels.

[49] Cervetto C., Amaroli A., Amato S., Gatta E., Diaspro A., Maura G., Signore A., Benedicenti S., Marcoli M. (2023). Photons Induce Vesicular Exocytotic Release of Glutamate in a Power-Dependent Way. Int. J. Mol. Sci..

[50] Schindelin J., Arganda-Carreras I., Frise E., Kaynig V., Longair M., Pietzsch T., Preibisch S., Rueden C., Saalfeld S., Schmid B. (2012). Fiji: An open-source platform for biological-image analysis. Nat. Methods.

[51] Pirnat S., Božić M., Dolanc D., Horvat A., Tavčar P., Vardjan N., Verkhratsky A., Zorec R., Stenovec M. (2021). Astrocyte arborization enhances Ca^2+^ but not cAMP signaling plasticity. Glia.

[52] Haseleu J., Anlauf E., Blaess S., Endl E., Derouiche A. (2013). Studying subcellular detail in fixed astrocytes: Dissociation of morphologically intact glial cells (DIMIGs). Front. Cell. Neurosci..

[53] Reversi A., Rimoldi V., Marrocco T., Cassoni P., Bussolati G., Parenti M., Chini B. (2005). The oxytocin receptor antagonist atosiban inhibits cell growth via a “biased agonist” mechanism. J. Biol. Chem..

[54] Passoni I., Leonzino M., Gigliucci V., Chini B., Busnelli M. (2016). Carbetocin is a Functional Selective Gq Agonist That Does Not Promote Oxytocin Receptor Recycling After Inducing β-Arrestin-Independent Internalisation. J. Neuroendocrinol..

[55] Alberi S., Dreifuss J.J., Raggenbass M. (1997). The oxytocin-induced inward current in vagal neurons of the rat is mediated by G protein activation but not by an increase in the intracellular calcium concentration. Eur. J. Neurosci..

[56] Cassoni P., Sapino A., Stella A., Fortunati N., Bussolati G. (1998). Presence and significance of oxytocin receptors in human neuroblastomas and glial tumors. Int. J. Cancer.

[57] Wiegand V., Gimpl G. (2012). Specification of the cholesterol interaction with the oxytocin receptor using a chimeric receptor approach. Eur. J. Pharmacol..

[58] Insel P.A., Ostrom R.S. (2003). Forskolin as a tool for examining adenylyl cyclase expression, regulation, and G protein signaling. Cell. Mol. Neurobiol..

[59] Burns D.L. (1988). Subunit structure and enzymic activity of pertussis toxin. Microbiol. Sci..

[60] Yamane H.K., Fung B.K. (1993). Covalent modifications of G-proteins. Annu. Rev. Pharmacol. Toxicol..

[61] Baudon A., Clauss Creusot E., Althammer F., Schaaf C.P., Charlet A. (2022). Emerging role of astrocytes in oxytocin-mediated control of neural circuits and brain functions. Prog. Neurobiol..

[62] Wettschureck N., Offermanns S. (2005). Mammalian G proteins and their cell type specific functions. Physiol. Rev..

[63] Montana V., Ni Y., Sunjara V., Hua X., Parpura V. (2004). Vesicular glutamate transporter-dependent glutamate release from astrocytes. J. Neurosci..

[64] Rothstein J.D., Martin L., Levey A.I., Dykes-Hoberg M., Jin L., Wu D., Nash N., Kuncl R.W. (1994). Localization of neuronal and glial glutamate transporters. Neuron.

[65] Guček A., Vardjan N., Zorec R. (2012). Exocytosis in astrocytes: Transmitter release and membrane signal regulation. Neurochem. Res..

[66] Vardjan N., Zorec R. (2015). Excitable Astrocytes: Ca^2+^- and cAMP-Regulated Exocytosis. Neurochem. Res..

[67] Tapia R., Sitges M. (1982). Effect of 4-aminopyridine on transmitter release in synaptosomes. Brain Res..

[68] Segovia G., Porras A., Mora F. (1997). Effects of 4-aminopyridine on extracellular concentrations of glutamate in striatum of the freely moving rat. Neurochem. Res..

[69] Agulhon C., Petravicz J., McMullen A.B., Sweger E.J., Minton S.K., Taves S.R., Casper K.B., Fiacco T.A., McCarthy K.D. (2008). What is the role of astrocyte calcium in neurophysiology?. Neuron.

[70] Xin W., Schuebel K.E., Jair K.W., Cimbro R., De Biase L.M., Goldman D., Bonci A. (2019). Ventral midbrain astrocytes display unique physiological features and sensitivity to dopamine D2 receptor signaling. Neuropsychopharmacology.

[71] Gravati M., Busnelli E., Bulgheroni A., Reversi P., Spaiardi M., Parenti M., Toselli B., Chini B. (2010). Dual modulation of inward rectifier potassium currents in olfactory neuronal cells by promiscuous G protein coupling of the oxytocin receptor. J. Neuro-chem..

[72] Stoop R. (2012). Neuromodulation by Oxytocin and Vasopressin. Neuron.

[73] Ayar A., Ozcan M., Alcin E., Serhatlioglu I., Ozcan S., Kutlu S., Kelestimur H. (2014). Oxytocin activates calcium signaling in rat sensory neurons through a protein kinase C-dependent mechanism. J. Physiol. Biochem..

[74] Eliava M., Melchior M., Knobloch-Bollmann H.S., Wahis J., da Silva Gouveia M., Tang Y., Ciobanu A.C., Triana Del Rio R., Roth L.C., Althammer F. (2016). A new population of parvocellular oxytocin neurons controlling magnocellular neuron activity and inflammatory pain processing. Neuron.

[75] Amzica F. (2002). In vivo electrophysiological evidences for cortical neuron–glia interactions during slow (<1 Hz) and paroxysmal sleep oscillations. J. Physiol. Paris.

[76] Dallérac G., Chever O., Rouach N. (2013). How do astrocytes shape synaptic transmission? Insights from electrophysiology. Front. Cell. Neurosci..

[77] Amzica F., Massimini M. (2002). Glial and neuronal interactions during slow wave and paroxysmal activities in the neocortex. Cereb. Cortex.

[78] Cahill M.K., Collard M., Tse V., Reitman M.E., Etchenique R., Kirst C., Poskanzer K.E. (2024). Network-level encoding of local neurotransmitters in cortical astrocytes. Nature.

[79] Chini B., Verhage M., Grinevich V. (2017). The Action Radius of Oxytocin Release in the Mammalian CNS: From Single Vesicles to Behavior. Trends Pharmacol. Sci..

[80] Tirko N.N., Eyring K.W., Carcea I., Mitre M., Chao M.V., Froemke R.C., Tsien R.W. (2018). Oxytocin Transforms Firing Mode of CA2 Hippocampal Neurons. Neuron.

[81] Kombian S.B., Hirasawa M., Mouginot D., Pittman Q.J. (2002). Modulation of synaptic transmission by oxytocin and vasopressin in the supraoptic nucleus. Prog. Brain Res..

[82] Osako Y., Otsuka T., Taniguchi M., Oka T., Kaba H. (2000). Oxytocin Depresses Spontaneous γ-Aminobutyric Acid-Ergic Inhibitory Postsynaptic Currents in Cultured Mitral Cells of the Rat Olfactory Bulb by a Presynaptic Mechanism. Neurosci. Lett..

[83] Hirasawa M., Kombian S.B., Pittman Q.J. (2001). Oxytocin Retrogradely Inhibits Evoked, but Not Miniature, EPSCs in the Rat Supraoptic Nucleus: Role of N- and P/Q-Type Calcium Channels. J. Physiol..

[84] Liu X.Y., Ling S., Liu Y., Jiang Y., Wang X., Jia S., Hou C., Li Y., Qiu D., Wang Y.F. (2025). G Proteins-Associated Dose-Dependent Effects of Oxytocin on Oxytocin Neuronal Activity and Astrocytic Plasticity of the Supraoptic Nucleus. Mol. Neurobiol..

[85] Choi H.B., Gordon G.R., Zhou N., Tai C., Rungta R.L., Martinez J., Milner T.A., Ryu J.K., McLarnon J.G., Tresguerres M. (2012). Metabolic communication between astrocytes and neurons via bicarbonate-responsive soluble adenylyl cyclase. Neuron.

[86] Kim S.H., MacIntyre D.A., Hanyaloglu A.C., Blanks A.M., Thornton S., Bennett P.R., Terzidou V. (2016). The oxytocin receptor antagonist, Atosiban, activates pro-inflammatory pathways in human amnion via G_αi_ signalling. Mol. Cell Endocrinol..

[87] Van Den Herrewegen Y., Sanderson T.M., Sahu S., De Bundel D., Bortolotto Z.A., Smolders I. (2021). Side-by-side comparison of the effects of Gq- and Gi-DREADD-mediated astrocyte modulation on intracellular calcium dynamics and synaptic plasticity in the hippocampal CA1. Mol. Brain.

